# Exercise and cancer-related lymphedema in the lower limbs—a randomized cross-over trial on high-intensity interval training (HIIT) with and without compression garments

**DOI:** 10.1007/s00520-025-09458-x

**Published:** 2025-04-16

**Authors:** Merete Celano Wittenkamp, Carsten Bogh Juhl, Bo Zerahn, Anders Vinther

**Affiliations:** 1 Department of Physiotherapy and Occupational Therapy, Copenhagen University Hospital, Herlev and Gentofte, Copenhagen, Denmark; 2 Research Unit of Musculoskeletal Function and Physiotherapy, Department of Sports Science and Clinical Biomechanics, University of Southern Denmark, Copenhagen, Denmark; 3 Department of Clinical Physiology and Nuclear Medicine, Copenhagen University Hospital, Herlev and Gentofte, Copenhagen, Denmark; 4 Department of Clinical Medicine, University of Copenhagen, Copenhagen, Denmark

**Keywords:** Cancer related lymphedema, Lower limb lymphedema, Compression, Exercise, High-intensity interval training

## Abstract

**Purpose:**

The aim was to evaluate the safety and possibility of performing high-intensity interval training (HIIT) on a stationary bike for participants with cancer-related lower limb lymphedema (LLL) with and without compression garments in a cross-over design.

**Methods:**

Twenty-one participants with LLL were randomized to two sessions of HIIT on a stationary bike, one with and one without compression garments. The sessions were separated by a seven-day washout period. The trial was carried out in a hospital setting from September to November 2018.

The acceptability and safety of the intervention were assessed. The safety was evaluated as adverse events and immediate and 24-h changes in self-reported symptoms (pain, heaviness, and tension). Additionally, recruitment, completion rate, and post-exercise changes in LLL were assessed by circumferential measurements of the legs, dual energy X-ray absorptiometry (DXA), and bioimpedance spectroscopy (BIS), respectively.

**Results:**

Twenty-one out of 35 (60%) eligible patients were included, and 19 (90%) patients completed both exercise sessions. Acceptability was high, and there were no adverse events. There was no clinically relevant difference between performing exercise with and without compression in self-reported symptoms or in limb volume. Small statistically significant differences in soft tissue mass (164.2 g corresponding to 1.4%) and extracellular fluid (L-Dex range < 5 units) were observed with and without compression, respectively, both favoring exercise with compression.

**Conclusion:**

HIIT on a stationary bike was acceptable for patients with LLL and seemed safe regardless of the use of compression garments.

**Trial registration:**

Clinicaltrials.gov registration (NCT03653819).

## Introduction

Lymphedema (LE) is a chronic condition due to impairment of the lymphatic system which causes accumulation of fluid in the subcutaneous tissue [1]. Primary LE is caused by congenital malformations, while cancer-related LE can develop due to lymph node removal. The risk of LE is increased by radiotherapy, infections, cellulitis, and obesity [2]. The incidence of breast cancer related LE after axillary node dissection is 20% [3], whereas the incidence of lower limb LE (LLL) is 28% in malignant melanoma, 20% in gynecological cancer, and 10% in urogenital cancers [2]. International consensus on the diagnostic criteria for LE is lacking, and as a consequence, the incidence of cancer-related LE should be interpreted with caution [2].

LE is characterized by visible swelling, heaviness, and tension and can also be painful. In later stages of LE, chronic skin changes occur with a risk of wounds and cellulitis [4]. LE can impair physical functioning, and previous studies have shown that cancer survivors with LE report lower quality of life [5, 6]. LE can be managed conservatively by complete decongestive therapy (CDT) consisting of skin care, bandaging, exercise, and manual lymphatic drainage, but the impairment of the lymphatic system is irreversible, demanding life-long self-management [1].

Despite the recommendation to exercise, patients with LE do not necessarily consider exercise as a part of their lymphedema self-management program [7]. Dunberger et al. [5] found that 27% in the subgroup of 218 patients with LLL did not adhere to regular exercise, and 77% reported that walking had a negative impact on LLL. Patients with LE also raise concern about whether exercise may exacerbate LE when seeking advice on exercise from health care professionals [7].

Exercise for patients with LE is safe and may decrease the symptoms from LE according to systematic reviews [8–11]. This is in line with guidelines on exercise for cancer survivors with LE from the American College of Sports Medicine [12]. However, most reviews did not include randomized controlled exercise studies on patients with LLL. Further, it is questionable whether conclusions based on studies on patients with upper limb lymphedema (ULL) apply to patients with LLL. Previously, a cross-over trial has examined the immediate effects on volume in patients with LLL after a 15-min exercise session on a bicycle ergometer with high load and low load intensity compared to no exercise [13]. Participants experienced a small decrease in limb volume after exercising, and biking seemed to be safe for participants with LLL [13]. In addition, biking appears to be suitable for patients with LLL since it engages a significant proportion of lower limb muscles without requiring full weight bearing.

The best practice document on LE management from the International Lymphedema Framework (ILF) recommends that patients with LE apply compression during exercise [1], which is supported by The National Lymphedema Network [14]. However, patients with LE may find it embarrassing or uncomfortable to wear compression garments during exercise [7], and this may prevent them from exercising. Singh et al. were only able to include four studies investigating the use of compression during exercise in patients with ULL in a systematic review and could not provide solid recommendations for the use of compression during exercise [8]. Consequently, the recommendations for the use of compression during exercise are not evidence-based.

The safety and ability to perform exercise for patients with LLL and the effect of compression during exercise remain to be investigated. Consequently, we performed a cross-over trial with high-intensity interval training (HIIT) on a stationary bike. The primary aim of our study was to investigate the acceptability and safety of the HIIT by adverse events and change in self-reported symptoms (pain, heaviness, and tension). The secondary aims were to explore recruitment, completion rate, and immediate effects on limb volume, soft tissue mass of the affected limb, and extracellular fluid in LLL.

## Methods

### Study design

Our study was designed as a cross-over trial and conducted in a hospital setting between September and November 2018. All participants completed two identical exercise sessions with a wash-out period of seven days in between, one session with made-to-measure compression garments, and one session without. To minimize daily variations in LE, both sessions were scheduled at the exact same time of day. Simple randomization of the sequence of exercise sessions with and without compression garments was performed using the app “Choose Random—Random Generator” from Google Play Store.

### Participants

We aimed to include 20 participants diagnosed with cancer-related LLL according to the following criteria: LE in stage I or II according to the classification from the International Society of Lymphology (ISL) [15], age > 18 years, daily use of made-to-measure compression garments, CDT completed, no ongoing oncologic treatment, and no known metastases. The participants were recruited from Departments of Physiotherapy at hospitals in the Capital Region or Region Zealand in Denmark, compression supply clinics, and by advertisement on the Facebook page of DALYFO (the Danish patient association for patients with LE). Pre-screening for eligibility was performed by telephone interview. After written consent and baseline measurements, eligibility was confirmed by checking patient records.

### Baseline

One to two weeks prior to the exercise sessions, participants completed the Graded Cycling Test with Talk Test (GCT-TT) as described by Nielsen et al. [16]. GCT-TT is an incremental exercise test performed on a stationary bike (Monark 928E from Monark Exercise, Vansbro, Sweden). The test is based on self-perceived exertion and is ended when the participant is no longer able to recite a standard text without forced pauses due to excessive breathing [16]. This exercise intensity corresponds to approximately 15–16 on the Borg scale [17] or an exercise intensity of approximately 60–84% of maximum oxygen uptake (VO_2_ max) [18]. The power output during the last minute of the test constitutes the test result [16]. Previous studies have shown that GCT-TT is reliable for patients with ischemic heart disease, minor stroke, and men with metastatic prostate cancer [16, 19, 20]. All participants wore their own made-to-measure compression garments during the GCT-TT.

### Intervention

**Fig. 1:**
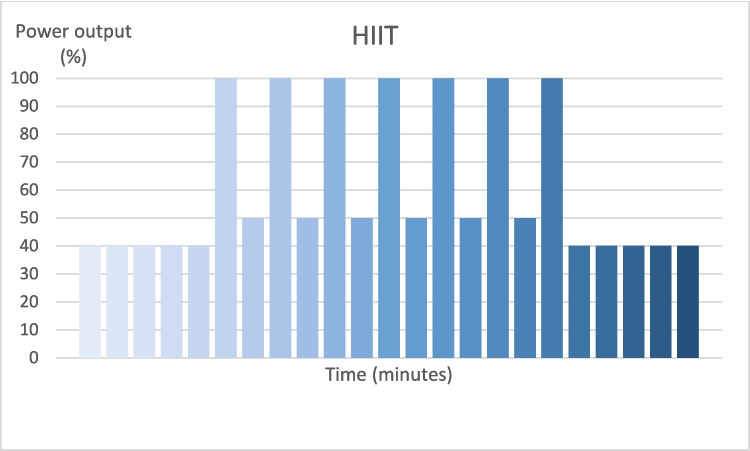
Illustration of the HIIT program

The intervention consisted of high-intensity interval training (HIIT) performed on a stationary bike in a hospital setting and supervised by an experienced physiotherapist. A previous study has shown that HIIT on a stationary bike is safe for cancer survivors [21]. The intervals in the HIIT in the studies by Toohey et al. lasted only 30 s [21, 22]. However, pretesting of the HIIT program showed that it took 5 to eight seconds before the load on the bike was adjusted to the entered workload, for which reason intervals with a duration of 1 min were chosen. Participants performed a 5-min warm-up and a 5-min cool-down with optional cadence. The workload on the bike was kept at approximately 40% of the final power output in the GCT-TT during warm-up and cool-down. The HIIT program consisted of seven intervals of 1 min of pedaling with the power output reached in the final minute of the GCT-TT (Fig. 1).

The relative exercise intensity during the high-intensity interval training (HIIT) program is shown on the *Y*-axis in percent of the intensity that was reached during the final stage of the submaximal cycling test (Graded Cycling Test with Talk Test) by each individual participant. The *X*-axis represents a total exercise time of 23 min—each bar representing 1 min.

Between intervals, the power output was reduced to approximately 50% of the final GCT-TT level, corresponding to an exercise intensity of 10–11 on the Borg scale. The cadence was kept as close to 80 revolutions per minute (rpm) as possible during the exercise session. Between exercise sessions, participants were asked not to change their routine use of compression garments and other compression regimes such as intermittent pneumatic compression device (IPC), self-bandaging, or nighttime compression. The participants were also asked not to change their physical activity, diet, or alcohol intake.

### Outcome

Acceptability and safety were assessed as primary outcomes, while recruitment, completion rate, and immediate effects on LLL were secondary outcomes. Acceptability was measured on a 4-point Likert scale, and good acceptability was a priori defined as > 75% of participants reporting to be “satisfied” or “very satisfied” with the intervention. Safety was assessed in terms of adverse events, such as cellulitis, skin irritation or wounds caused by compression garments, and changes in self-reported pain, heaviness, and tension measured on a numerical rating scale (NRS) after exercising and 24 h post-exercise. Data collection of self-reported symptoms, acceptability, and adverse events was performed by self-administered electronic surveys.

Measurements of limb volume, soft tissue mass of the affected limb, and extracellular fluid were collected pre- and post-exercise. The volume was estimated by circumferential measurements (CM) with a Gulick tape at 8-cm intervals following a standard protocol and converted to volume using the formula for truncated cone as recommended by Hidding et al. [23]. Dual energy X-ray absorptiometry (DXA) was used for assessment of soft tissue mass of the affected limb. Full body scans (Lunar Prodigy, GE Medical Systems, Madison, WI) were performed followed by manual drawing of regions of interest (ROI) of the thigh and lower leg to calculate segmental body composition of the legs (bone mineral content (BMC), fat mass, and soft tissue mass) in grams as previously described by Gjorup et al. [24]. Mean changes in soft tissue mass were expected to be due to changes in LLL. The changes in BMC for thigh and lower limb after each session were assessed and compared to limits of agreements (LOA) from Gjorup et al. [24] to confirm the precision of all ROIs. The foot was excluded in the analysis because of the moderate reliability of soft tissue mass of the foot as reported by Gjorup et al. [24]. The amount of extracellular fluid was measured by bioimpedance spectroscopy (BIS) and described by the L-Dex score (Lymphedema inDex), a ratio score comparing the amount of extracellular fluid between the limbs for unilateral cases or right arm/leg versus left arm/leg for bilateral cases (SOZO, Impedimed). Among bilateral cases, the largest limb as measured by baseline CM was categorized as the affected limb in the analysis of data from CM, DXA and BIS.

### Statistical analysis

Participants’ characteristics are described by mean and standard deviations for continuous variables and by number and percent for categorical variables. Differences in changes between exercise sessions with and without compression were evaluated by paired *t*-tests for the following outcomes: (1) self-reported pain, heaviness, and tension; (2) lean mass of the thigh and lower leg measured by DXA; (3) volume calculations in ml; and (4) L-Dex Scores from BIS. The statistical significance level was set at *p* < 0.05, and analyses were performed in STATA (version 18).

## Results

### Participant characteristics

Eighteen women and three men with a mean age of 52.1 years (SD 12.3) and a mean BMI of 25.0 (SD 3.7) were included, 15 of whom had unilateral LLL. Baseline characteristics are presented in Table 1. Most participants had stage I (23.8%) or early stage II LLL (61.9%) according to staging from ISL. Mean volume difference between the limbs was 666.5 ml (SD 780.8) in unilateral cases and 336.2 ml (SD 270.9) for participants with bilateral LLL. Most participants wore pantyhose or thigh-length stockings, and most participants used compression class III (Table 1).

**Table 1:** Characteristics of the enrolled participants (*n* = 21)

Variable	*n* (%) or mean (SD)
Gender (women)	18 (86.0%)
Age (years)	52.1 (12.3)
Body mass index (kg/m^2^)	25.0 (3.7)
Time since cancer diagnosis (years)	6.0 (8.5)
Cancer type	
Gynecological cancer	15 (71.4%)
Melanoma	4 (19.0%)
Urogenital cancer	2 (9.5%)
Lymph nodes removed^a^	22.2 (11.9)
Adjuvant oncologic treatment	
Chemotherapy	7 (33.3%)
Radiotherapy	5 (23.8%)
Time since diagnosis of lymphedema (years)	4.3 (6.6)
Stage of lymphedema (ISL)^b^	
Stage I	5 (23.8%)
Early stage II	13 (61.9%)
Late stage II	3 (14.2%)
Inter-limb volume difference	
Unilateral cases ml (range) *n* = 15	666.5 (780.8)
Bilateral cases ml (range) *n* = 6	336.2 (270.9)
Use of compression garments	
Daytime only	15 (71.4%)
Daytime + nighttime compression	6 (28.6%)
Type of compression garment	
Pantyhose	3 (14.2%)
Thigh length	10 (47.6%)
Thigh length + Bermuda	7 (33.3%)
Knee length	1 (4.8%)
Compression class^c^	
II (23–32 mmHg)	2 (9.5%)
III (34–46 mmHg)	19 (90.5%)
Use compression garments during exercise	
Always	11 (52.4%)
Occasionally	2 (9.5%)
Never	6 (28.6%)
Do not exercise	2 (9.5%)

Values are presented as *n* or mean (% or SD), unless otherwise indicated. *SD*, standard deviation. ^a^Data on number of lymph nodes removed were only available on 17 participants. ^b^Classification from the International Society of Lymphology (ISL). ^c^Compression garments are available in compression class I–IV. Compression classes are the measure of the pressure exerted on the ankle, presented in the unit; millimeters of mercury (mmHg)

### Recruitment and completion rate

**Fig. 2:**
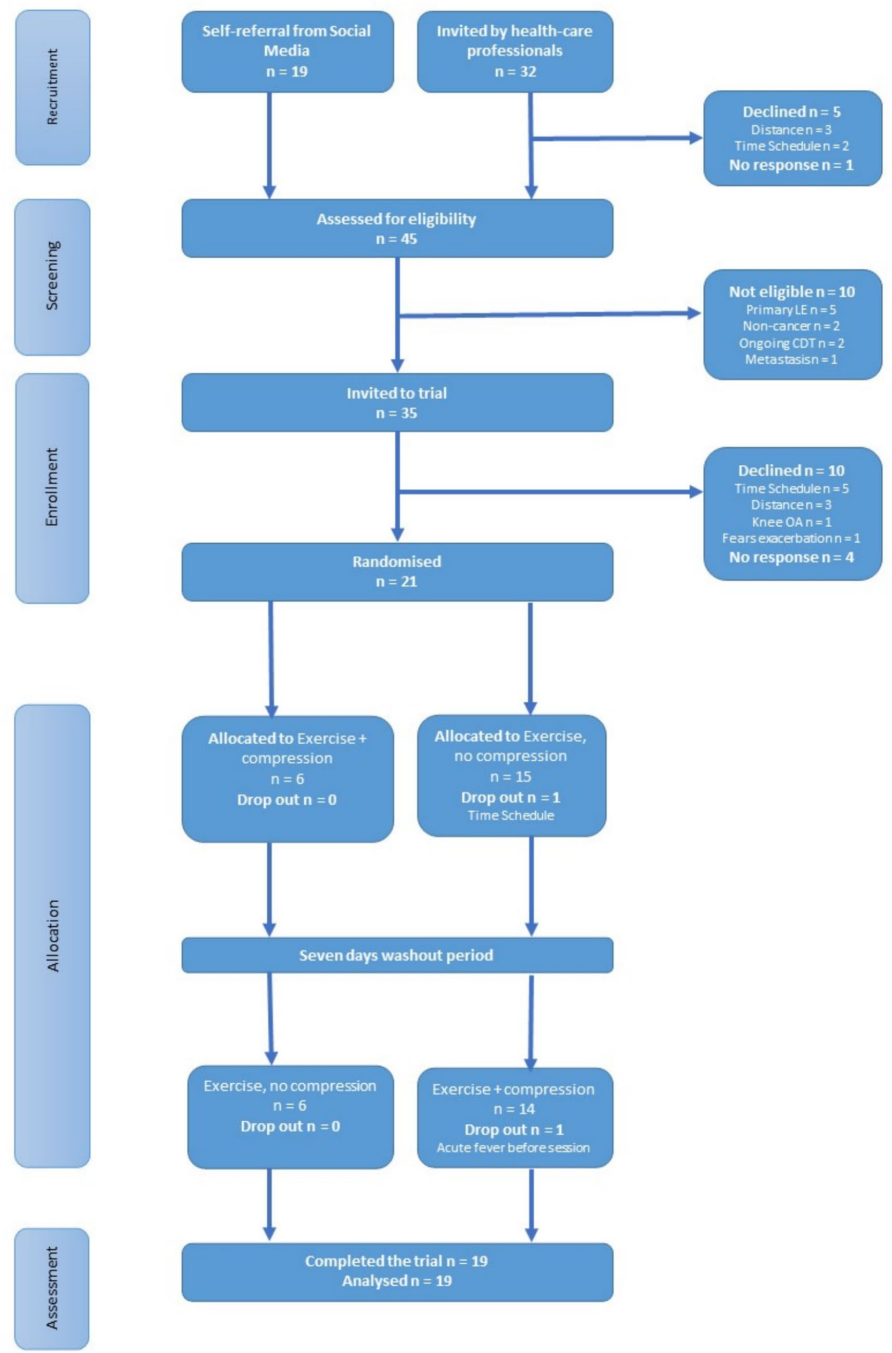
Flowchart of recruitment and completion of the trial

A total of 45 participants were screened for eligibility by telephone by health care professionals and were informed about the trial. Ten participants did not meet the inclusion criteria, leaving 35 eligible participants who received written information about the trial. Fourteen patients declined participation (Fig. 2).

A total of 21 participants were included (60% of the eligible participants), but one participant withdrew after randomization due to the time schedule. Six participants were randomized to exercise with compression garments in the first session, and 15 participants were randomized to exercise without compression garments in the first session.

One participant dropped out before the second exercise session due to acute fever. A total of 19 participants (90% of the allocated participants) completed the intervention. However, three participants were unable to hold the cadence of 80 rpm in the HIIT program and a power output corresponding to the final minute of their GCT-TT (Table 2).

**Table 2:** Completion and acceptability of the trial

**Completion of the GCT-TT**		
Completed GCT-TT	21 (100%)	
Final power out (watt) mean (SD)	127.1 (22.6)	
**Completion of the HIIT program**	**No compression** (*n* = 20)	**Compression** (*n* = 19)
No adjustments	17 (85.0%)	16 (84.2%)
With adjustments	3 (15.0%)	3 (15.8%)
Serious adverse events	0 (0.0%)	0 (0.0%)
**Acceptability of the HIIT program**		
Very satisfied	18 (90.0%)	15 (78.9%)
Satisfied	0 (0.0%)	3 (15.8%)
A bit dissatisfied	1 (5.0%)	0 (0.0%)
Very dissatisfied	1 (5.0%)	1 (5.3%)
Concern about exacerbation of lymphedema	1 (5.0%)	0 (0.0%)
**Preference for compression in the trial** (*n* = 19)		
Without compression	8 (42.1%)	
With compression	8 (42.1%)	
In doubt	3 (15.8%)	

Values are presented as *n* (%) unless otherwise indicated. *SD*, standard deviation; *GCT-TT*, Graded Cycling Test with Talk Test

Consequently, the target power outputs during both exercise sessions were reduced for these three participants by 10, 20, and 25 W, respectively.

### Acceptability

Most participants were “satisfied” or “very satisfied” with the intervention, 18 of 20 for the session without compression and 18 of 19 for the session with compression (Table 2). Before the exercise, one participant expressed concern about exercising without compression. After completion, eight participants favored exercising with compression garments, while eight participants were inclined towards exercising without. Three participants had no preference (Table 2). Most participants emphasized the comfort and unrestricted range of movement experienced during the exercise session without compression garments. Participants with pantyhose or a combination of Bermuda and thigh-length compression stockings expressed discomfort, such as muscle weakness and heaviness in the affected limbs, during the exercise session with compression garments.

### Safety

#### Adverse events

No adverse events were reported during the trial (Table 2). Two participants reported delayed onset muscle soreness (DOMS) after the first exercise session, but both were able to complete both sessions.

#### Self-reported outcome measures

No difference in mean change in self-reported pain, heaviness, and tension was found between exercising with or without compression immediately after exercise: 0.4 (95% CI − 0.1; 0.8), 0.0 (95% CI − 0.8; 0.8), and 0.5 (95% CI − 0.2; 1.1), respectively, nor at 24 h follow-up: − 0.3 (95% CI − 1.2; 0.6), 0.1 (95% CI − 1.1; 1.3), and − 0.1 (95% CI − 1.1; 0.9), respectively (Table 3).

**Table 3:** Changes in NRS scores, volume, soft tissue mass, and extracellular fluid

	Mean change (95% CI)		Difference mean change(95% CI)	Between sessions *p*-value
	Compression	No compression		
**Self-reported outcome measures (NRS)**				
*Post-exercise*				
Pain	− 0.1 (− 0.4, 0.3)	− 0.4 (− 0.8, − 0.1)	0.4 (− 0.1, 0.8)	0.09
Heaviness	− 0.2 (− 0.8, 0.5)	− 0.2 (− 0.9, 0.6)	0.0 (− 0.8, 0.8)	1.00
Tension	− 0.1 (− 0.8, 0.7)	− 0.5 (− 1.3, 0.3)	0.5 (− 0.2, 1.1)	0.2
*24-h post-exercise*				
Pain	− 0.2 (− 0.4, 0.1)	0.2 (− 0.6, 0.9)	− 0.3 (− 1.2, 0.6)	0.5
Heaviness	0.2 (− 0.4, 0.8)	0.1 (− 0.8, 1.1)	0.1 (− 1.1, 1.3)	0.2
Tension	0.0 (− 0.6, 0.6)	0.1 (− 0.6, 0.9)	− 0.1 (− 1.1, 0.9)	0.3
**Objective outcome measures**				
Affected limb				
Volume (ml)	65.3 (− 24.6, 155.2)	83.9 (− 13.4, 181.3)	− 18.7 (− 150.2, 112.8)	0.8
Soft tissue mass (g)	26.6 (− 47.2,100.4)	190.8 (77.7, 303.9)	− 164.2 (− 298.2, − 30.2)	0.02
Extracellular fluid (L-Dex)	− 2.5 (− 7.4, 2.4)	2.3 (− 0.3, 4.8)	− 4.8 (− 9.1, − 0.5)	0.03

*N* = 19. *CI*, confidence interval; *NRS*, numeric rating scale. Volume calculated into milliliters (ml) by formula of truncated cone from circumferential measurements. Soft tissue mass assessed by drawing of regions of interest after full body scan with dual energy X-ray absorptiometry scan. Extracellular fluid measured as L-Dex by bioimpedance spectroscopy. For participants with bilateral lower limb lymphedema, L-Dex values from the most affected limb were used for calculation of mean change scores

#### Objective outcome measures

The mean change in soft tissue mass after exercise without compression was statistically significant: + 190.8 g (95% CI 77.7; 303.9, *p*-value: 0.002), whereas no significant difference was found in self-reported outcomes (pain, volume, and heaviness) and other objective measures (extracellular fluid and volume). Statistically significant differences in mean change in soft tissue mass (g) and extracellular fluid (L-Dex) were found between exercising with or without compression after exercise: − 164.2 (95% CI − 298.2, − 30.2), and − 4.8 (95% CI − 9.1; − 0.5), respectively, both in favor of exercising with compression (Table 3). No difference in mean change in volume (ml) was found after exercise: − 18.7 (95% CI − 150.2: 112.8).

## Discussion

Acceptability was high, and no serious adverse events occurred during HIIT with and without compression garments in participants with LLL. A recruitment rate with an allocation of 60% of the eligible participants and a completion rate of 90% confirmed the possibility to run the trial. The differences in changes in soft tissue mass and extracellular fluid between sessions with and without compression were statistically significant in favor of compression garments. However, the findings did not translate into differences in self-reported symptoms or changes in volume, respectively. Minimal Important Clinical Difference (MCID) has not been defined for neither change in soft tissue mass nor L-Dex in patients with lower limb lymphedema to help the interpretation of the significant differences. The observed larger increase in soft tissue mass of 164.2 g corresponds to 1.4% and change in extracellular fluid with L-Dex < 5 units. Vicini et al. have suggested a threshold for a change in L-Dex at 7 units or above for early diagnostics of subclinical lymphedema in breast-cancer patients [25], but no thresholds have been established for either early diagnostics or evaluation in lower limb lymphedema as the SOZO technology is a fairly new research entity. As such, we have chosen to interpret the significant results with caution and consider them small and not clinically important. Additionally, the re-application of compression garments after exercise provides external graduated pressure to the limb, which will support the lymphatic system with an expected decrease in LLL post-exercise. The hypothesis is supported by the lack of increase in self-reported LLL symptoms at 24 h and similar results with a decrease in volume 24 h post-exercise in trials for upper limb lymphedema [26, 27]. Consequently, the results confirm that HIIT performed on a bike is safe for participants with LLL, both with and without compression garments.

There is a risk of selection bias in the study since a trial with HIIT may appeal to participants with a moderate or high level of fitness. Data were not collected on eligible participants who declined participation, but the participants in our trial were relatively young and had a low BMI and low volume of the affected limb compared to participants with LLL in other exercise trials [28–32]. The risk of selection bias may well be accentuated because of recruitment via social media. Therefore, the allocation rate must be expected to be lower in future trials with consecutive recruitment at hospitals.

Another limitation in our study is the lack of blinding. Obviously, participant blinding of the use of compression garments was not possible, while blinding of the assessor might have strengthened the study. However, markings from compression garments will appear on the skin of the limbs affected by LE and thereby invalidate the blinding since assessments were performed immediately after the exercise sessions in this trial. Moreover, blinding of the assessor is not likely to affect the results of the patient reported outcomes, nor of DXA-scans and BIS. In addition, the randomization process by the app resulted in an unbalanced allocation, but since participants acted as their own controls in our cross-over trial, the allocation is unlikely to affect the results.

All tests and data collection were carried out by the same experienced physiotherapist to reduce the risk of measurement error. All measurements were performed in the same order before and after each exercise session and within 75 min. The washout period of 1 week was appropriate since it included the same number of workdays and a weekend for all participants regardless of which day of the week the exercise session was performed. Moreover, 1 week was also chosen as washout period in earlier cross-over exercise trials on patients with LLL [13, 33]. Additionally, participants were scheduled to exercise at the same time of the day on both sessions to minimize variation in LLL during the day.

Water volumetry is the “Gold Standard” for volume assessment in LE and has excellent reliability [23]. However, the water volumetry method is cumbersome and requires strict hygiene procedures. Instead, the volume of the limbs was calculated from circumferential measurements using the formula for a truncated cone. A Gulick Tape was used, and a standard protocol was followed to increase the reliability and validity of the measurements as recommended by Hidding et al. [23]. DXA scans have been found to be superior to water volumetry and CM in assessment of LE [34]. The regions of interest (ROI) were drawn manually as described by Gjorup et al. [24], who reported an intraclass correlation coefficient (ICC) between 0.97 and 0.99 for both volume and tissue composition of the thigh and lower leg with narrow limits of agreement (LOA). Thus, the precision of the ROIs is expected to be high, which may explain significant changes measured by DXA was not captured by CM or self-report. BIS is recommended as a screening and diagnostic tool for subclinical LE and stage I LE compared to circumferential measurements and water volumetry [23]. Turgay et al. did not find any correlation between BIS and LE severity [35]. Therefore, the validity of BIS as an outcome measure in our trial with participants diagnosed with LE in late stage II may be questionable since BIS measures the amount of extracellular fluid, which is less prevalent in late stages of LE when tissue fibrosis develops [15, 36].

The short duration of the HIIT and the individually adjusted exercise intensity based on pre-intervention assessment of aerobic exercise capacity (GCT-TT) may explain the high completion rate and acceptability among participants. GCT-TT was tolerated well by all participants, and the Talk Test seemed appropriate for estimating power output corresponding to a level of 15–16 on the Borg scale for the HIIT program in our trial. An exercise session with HIIT of only 23 min duration was also expected to be safe and to ensure completion of sessions regardless of fitness level among the participants while still inducing an adequate exercise stimulus on the lymphatic system.

Two cross-over trials with 15-min exercise sessions performed on a bicycle ergometer with the application of compression bandaging (CB) in participants with LLL have shown significant decrease in limb volume compared to CB alone [13, 33]. This effect of exercise in combination with compression was not seen in this study. This may be explained by insufficient pressure compared to newly applied compression bandages with a high sub-bandage pressure in the trials by Fukushima et al. and Abe et al. [13, 33], but the sub-garment pressure was not measured in our trial. Barufi et al. also reported an increase in LLL after 1-h sessions of walking with compression garments with insufficient pressure or no compression. The study reported a decrease in LLL when participants walked with a well-fitted compression garment [37]. Unfortunately, neither our trial on HIIT nor the trial on walking did a re-assessment of LLL after 3–4 h or 24 h to investigate if the immediate increase in LLL was reversed. Such follow-up procedures might have further strengthened the study results.

### Implications for practice and clinical research

In summary, the findings of this study show that it was possible to recruit participants with LLL to an exercise intervention with HIIT. Additionally, no serious adverse events occurred, and the immediate increase in soft tissue mass and extracellular fluid after exercise without compression garments is expected to normalize with reapplication of compression based on previous studies trials [26, 27]. However, the results are only based on 2 sessions of HIIT, and the long-term effect on LLL symptoms and objective outcomes was not investigated. Larger and longitudinal exercise trials in the field of LLL are clearly needed.

Several participants felt uncomfortable when exercising with Bermuda in combination with thigh-high stockings. They reported higher rates of exertion during the exercise and were among the participants who preferred to exercise without compression. Participants with limited volume increase and severity of LLL may benefit from sports compression tights. Sports compression clothing is widely used among athletes to improve performance and recovery time and reduce muscle soreness [38]. A pilot study found a decrease in extracellular fluid in participants with stage I LLL measured by BIS, although perometry did not confirm the finding [39]. The authors suggested that sports compression tights might remove barriers towards engaging in exercise since sports compression tights can seem more socially acceptable than regular compression garments and are easier to apply.

Based on the findings from our trial, clinicians may consider a less conservative approach to the use of compression garments during exercise for patients with LLL since exercise often is of short duration and compression garments are reapplied after exercise. This may increase the adherence to regular exercise and improve the health and wellbeing of patients with LLL. However, advice on exercise and compression garments to patients with LLL should be individualized until evidence is provided as highlighted by Singh et al. [8].

## Data Availability

No datasets were generated or analysed during the current study.
